# Wymioty Jako Objaw Padaczki. Zespół Panayiotopoulosa - Przegląd Literatury i Doświadczenia własne

**DOI:** 10.34763/devperiodmed.20192301.2833

**Published:** 2019-04-08

**Authors:** Magdalena Tworkiewicz, Agnieszka Sakson-Słomińska, Renata Kuczyńska, Krzysztof Słomiński, Aneta Krogulska

**Affiliations:** 1 Klinika Pediatrii Alergologii i Gastroenterologii Collegium Medicum w Bydgoszczy, Uniwersytet Mikołaja Kopernika w Toruniu, Polska; 2 Centrum Interwencyjnego Leczenia Udarów Mózgu. Oddział Neurologii Szpitala Uniwersyteckiego nr 2 im. J. Biziela w Bydgoszczy, Polska

**Keywords:** wymioty, zespół padaczkowy, dzieci, vomiting, epileptic syndrome, children

## Abstract

Wymioty są często spotykanym objawem w populacji pediatrycznej. Ich etiologia jest zróżnicowana, począwszy od łagodnych zaburzeń czynnościowych, aż do ciężkich zagrażających życiu chorób układowych. Najczęściej występują w przebiegu chorób przewodu pokarmowego, ale mogą dotyczyć także wielu innych schorzeń zlokalizowanych poza nim. Z uwagi na zróżnicowaną etiologię obejmującą różne układy i narządy niekiedy mogą sprawiać trudności diagnostyczne. W niniejszym artykule przedstawiono przypadek zespołu Panayiotopou/osa – dziecięcej padaczki potylicznej o wczesnym początku (Early-onset childhood occipital epilepsy). Charakterystyczną cechą tego zespołu są napady z objawami ze strony układu autonomicznego lub występowanie autonomicznych stanów padaczkowych. Dominującymi objawami są wymioty i nudności, które w pierwszej kolejności najczęściej sugerują zapalenie żołądka czy jelit, bądź migrenę lub proces rozrostowy w ośrodkowym układzie nerwowym. Rzadko w diagnostyce różnicowej bierze się pod uwagę możliwość występowania wymiotów jako elementu napadu padaczkowego. Celem naszej pracy jest zwrócenie uwagi na trudności w zdefiniowaniu właściwej przyczyny nawracających wymiotów. Niejednokrotnie, pomimo szczegółowo zebranego wywiadu oraz szerokiej diagnostyki, dopiero bezpośrednie stwierdzenie charakterystycznych objawów dla danego zespołu pozwala na ustalenie ostatecznego rozpoznania.

## Abstract (pl)

Vomiting is a common sign of illness in the pediatrie popu/ation. Its etiology is diversified, ranging from mild functional disorders to severe life-threatening systemic diseases. Vomiting most often occurs in the course of gastrointestinal tract diseases, however, it may also coexist with numerous other ailments located outside the Gl tract. Due to its diverse etiology encompassing various systems and organs, it can sometimes cause diagnostic difficulties. The present paper illustrates a case of Panayiotopoulos syndrome, which is an early-onset childhood occipital epilepsy (EOCOE). Characteristic of this syndrome are seizures with symptoms originating from the autonomic nervous system or the occurrence of vegetative status epilepticus. The dominant signs and symptoms are vomiting and nausea, which in the first place most frequently suggest inflammation of the stornach or intestines, migraine, or a proliferative process in the central nervous system. Rarely is the possibility of vomiting taken into account as an element of epileptic seizure in the differential diagnosis. The aim of this paper is to draw attention to the difficulty in defining the precise cause of recurrent vomiting. Many times, despite collecting a detailed medical history and extensive physical examination, it is only observation-based diagnosis that allows the doctor to make a final evaluation.

## Wstęp

Wymioty są nieswoistym, często występującym u dzieci objawem, o zróżnicowanej etiologii, począwszy od łagodnych zaburzeń czynnościowych, aż do ciężkich zagrażających życiu chorób układowych. W większości przypadków problem ustępuje szybko i samoistnie. Wymioty najczęściej występują w przebiegu chorób przewodu pokarmowego, ale mogą dotyczyć także wielu innych schorzeń zlokalizowanych poza nim (tab. I). Etiologia wymiotów u dzieci jest związana z wiekiem (tab. II). Do podstawowych gastroenterologicznych przyczyn wymiotów u dzieci starszych należą: patologiczny refluks żołądkowo-przełykowy, zapalenie żołądka z zakażeniem *Helicobacter pylori*, zapalenie wątroby, zapalenie trzustki, ostre schorzenia chirurgiczne (zapalenie wyrostka robaczkowego, zapalenie otrzewnej) czy zespół cyklicznych wymiotów. W ustaleniu ich etiologii podstawowe znaczenie ma dokładnie zebrany wywiad zawierający pytania o początek, częstotliwość, czas trwania wymiotów oraz objawy towarzyszące [1,2].

**Tabela I:** Diagnostyka różnicowa nawracających nudności i wymiotów. Table I. Differential diagnosis of recurrent nausea and vomiting [1].

Leki i toksyny *Medications and toxins*	Infekcje *Infections*	Choroby przewodu pokarmowego *Gastrointestinal*	Choroby neuropsychiatryczne *Neuropsychiatric disorders*	Choroby układu moczowego *Renal*	Choroby metaboliczne i endokrynne *Metabolic and endocrine*
		• niedrożność jelit *bowel obstruction* – niedokonany zwrot jelit *malrotation with volvulus* – przepukliny uwięźnięte *incarcerated hernias*			
• antybiotyki *antibiotics*	• zakażenia żołądkowo-jelitowe	– torbiele *duplication cyst* • zmiany zapalne	• migrena *migraine* • padaczka *epilepsy*		• ciąża (u nastolatków) *pregnancy (in adolescents)* • cukrzyca *diabetes mellitus*
• NLPZ *NSAIDs*	*enteric agents* • zapalenie wątroby	*inflammatory* – choroba wrzodowa	• wodogłowie *hydrocephalus*		• choroba Addisona *Addison mellitus*
• wysokie dawki *witamin*	*hepatitis* • zakażenie EBV *infection Epstein*	*peptic ulcer disease* – z.bł.śluzowej żołądka	– guz tylnego dołu czaszki	• zwężenie pęcherzowo-moczowodowe	• guz chromochłonny *Phaeochromocytoma*
*high-dose vitamins* • leki hormonalne *hormonal preparations*	*Barr virus* • zapalenie ucha środkowego	*gastritis* – z.błony śluzowej dwunastnicy *duodenitis*	*posterior fossa tumors* – krwawienie podtwardówkowe	*vesico-ureteral obstruction* • kamica nerkowa *nephrolithiasis*	• kwasice organiczne *organic acidurias* • zaburzenia oksydacji kwasów tłuszczowych
• środki przeczyszczające *laxatives*	*otitis media* • przewlekłe zapalenie zatok	– zapalenie wyrostka robaczkowego *chronic appendicitis*	*subdural effusion* • stany lękowe *anxiety states*		*fatty acid oxidation defects* • zaburzenia mitochondrialne *mitochondrial disorders*
	*chronic sinusitis*	– nieswoiste zapalenia jelit *inflammatory bowel disease*	• bulimia *nervosa*		• zaburzenia cyklu mocznikowego *urea cycle defects*
		• zapalenia trzustki *pancreatitis*			
		• choroby wątroby i dróg żółciowych *hepatobiliary disease*			

**Tabela II:** Diagnostyka różnicowa wymiotów u dzieci w zależności od wieku. Table II. Differential diagnosis of vomiting in children according to the age [2].

Niemowlę *Infant*	Małe dziecko *Toddler*	Nastolatek *Adolescent*
		Zapalenie żołądka i jelit *Gastroenteritis*
Zatrucie pokarmowe	Zapalenie żołądka i jelit	Choroba refluksowa *GERD*
*Food poisoning*	*Gastroenterititis*	Zapalenie wyrostka
Zapalenie żołądka i jelt	Zapalenie ucha środkowego, gardła, zatok	*Appendictitis*
*Gastroenterititis*	*Otitis media, sinusitis, pharyngitis*	Zatrucie toksynami
Choroba refluksowa	ZUM	*Toxin ingestion*
*GERD*	*UTI*	Migrena
Przekarmianie	Zapalenie opon mózgowo-rdzeniowych	*Migraine*
*Overfeeding*	*Meningitis*	NZJ
Nietolerancje pokarmowe	Zatrucia lekami	*Inflammatory bowel disease*
*Food intolerance*	*Toxic Drugs*	Zapalenie wątroby
ZUM	Cukrzyca	*Hepatitis*
*UTI*	*Diabetes*	Wrzód trawienny
Zapalenie ucha środkowego	Migrena	*Peptic ulcers*
*Otitis media*	*Migraine*	Psychologiczne
Niedokonany zwrot jelit	Wrzód trawienny	*Psychological*
*Malrotation*	*Peptic ulcers*	Zapalenie trzustki
Kwasica kanalikowo-nerkowa	Zapalenie trzustki	*Pancreatitis*
*Renal Tubular Acidosis*	*Pancreatitis*	Zapalenie pęcherzyka żółciowego
Alergia na białka mleka krowiego	Zespół wzmożonego	*Cholelithiasis*
*Milk alergy*	ciśnienia śródczaszkowego	Kolka nerkowa
Niewydolność nerek	*Raised ICP*	*Renal colic*
*Renal insufficiency*	Zespół Reya	Zespół wzmożonego
Zespół wzmożonego	*Reyes syndrome*	ciśnienia śródczaszkowego
ciśnienia śródczaszkowego	Zespół cyklicznych wymiotów	*Raised ICP*
*Raised ICP*	*Cyclic vomiting syndrome*	Zapalenie ucha środkowego *Otitis media*
		Cykliczne wymioty *Cyclic Vomiting*

Celem pracy jest przedstawienie trudności diagnostycznych u pacjentki, u której przyczyną uporczywych wymiotów ostatecznie okazała się być padaczka. Po prawie 2 latach występowania nawracających wymiotów, kilkukrotnych hospitalizacjach, przeprowadzeniu wielokierunkowej diagnostyki, dopiero po zaobserwowaniu charakterystycznych objawów w trakcie pobytu w Klinice rozpoznano zespół Panayiotopoulosa, należący do padaczek o początku w dzieciństwie [3].

## Opis przypadku

Dziewczynka 11-letnia została przyjęta do Kliniki z powodu nawracających wymiotów i bólów brzucha celem diagnostyki i leczenia. Z wywiadu wiadomo, że sporadyczne wymioty pojawiły się u dziewczynki w 9 rż. W pierwszym roku ich występowania nie obserwowano cykliczności ani towarzyszących dolegliwości, natomiast w 10 rż. ich częstotliwość uległa nasileniu, a rodzice zaobserwowali, że wymioty poprzedzały: złe samopoczucie, lęk (dziewczynka była „wystraszona”, „jakby nieobecna”), zawroty głowy, senność oraz silny ból głowy. Najczęściej epizody trwały 1 dzień, ustępowały samoistnie. W ciągu miesiąca poprzedzającego hospitalizację w Klinice dolegliwości występowały średnio co tydzień. Wymiociny nigdy nie zawierały niepokojących domieszek, nie było konieczności wyrównywania zaburzeń jonowych oraz podaży płynów infuzyjnych.

Dziewczynka z tego powodu była wielokrotnie hospitalizowana w różnych ośrodkach w Polsce. W wyniku przeprowadzonej diagnostyki wykluczono: proces rozrostowy OUN w oddziale neurologii (w MRI głowy uwidoczniono niewielką torbiel szyszynki, nieodpowiadającą za obserwowane objawy, wymagającą jedynie okresowej kontroli neurochirurgicznej), zaburzenia gospodarki węglowodanowej w oddziale endokrynologii, boreliozę oraz padaczkę w kolejnym oddziale neurologii (na podstawie prawidłowego 24-godzinnego zapisu EEG nie potwierdzono padaczki). Dziewczynkę dwukrotnie konsultował psycholog, nie stwierdzając istotnych nieprawidłowości. Z wywiadu wynika również, że rodzicom kilkukrotnie sugerowano konieczność konsultacji psychiatrycznej. Poza tym rodzice zgłosili pojawianie się grudkowych, swędzących zmian skórnych w zgięciach łokciowych i na twarzy.

Przy przyjęciu do Kliniki stan dziecka był dobry. Z odchyleń od normy w badaniu przedmiotowym stwierdzono: pojedyncze zmiany grudkowe w zgięciach łokciowych. W badaniach laboratoryjnych bez nieprawidłowości. Ze względu na zgłaszane w trakcie epizodów zawroty głowy dziewczynka była konsultowana przez laryngologa, który nie potwierdził cech ostrego uszkodzenia części obwodowej zmysłu równowagi. Konsultujący psychiatra stwierdził możliwość występowania zachowań hipochondrycznych.

W kolejnym dniu hospitalizacji dziewczynka od rana zgłaszała pogorszenie samopoczucia i zawroty głowy. W godzinach popołudniowych, w pozycji leżącej, wystąpiły jakościowe zaburzenia świadomości pod postacią pogorszenia kontaktu logicznego, z towarzyszącymi automatyzmami w formie głośnego przełykania śliny utrzymujące się około 10 minut oraz wymioty. Następnie doszło do utraty przytomności i wystąpił uogólniony napad padaczkowy toniczno-kloniczny trwający około 2 minut. Po odzyskaniu przytomności obserwowano stopniowe ustępowanie ilościowych zaburzeń świadomości (senność ponapadową).

Na podstawie przebiegu klinicznego i otrzymanych wyników badań wysunięto podejrzenie zespołu Panayiotopoulosa. Dziewczynkę przekazano do Oddziału Neurologii Dziecięcej celem dalszej diagnostyki i leczenia. Ponownie wykonano EEG, w którym w stanie czuwania w odprowadzeniach potylicznych stwierdzono czynność napadową o charakterze iglic, w pozostałych odprowadzeniach, bez nieprawidłowości. Do leczenia włączono na stałe preparat kwasu walproinowego. Od tej pory, tj. przez kolejne 8 miesięcy, do dziś nie wystąpił żaden epizod drgawkowy, nie obserwowano zaburzeń świadomości ani wymiotów.

## Dyskusja

Zespół Panayiotopoulosa to dziecięca padaczka potyliczna o wczesnym początku [3]. Po raz pierwszy został opisany w 1989 r. przez Panayiotopoulosa [4], który jako charakterystyczne opisał triadę objawów, tj.: nocne napady padaczkowe-toniczne ze zwrotem gałek ocznych, wymioty oraz zmiany w zapisie EEG w okolicy potylicznej [5,6]. Kilka lat później zaproponowano nazwę łagodna dziecięca padaczka potyliczna o wczesnym początku, obecnie dziecięca padaczka potyliczna o wczesnym początku (EOCOE) [3, 7, 8]. Wg ekspertów głównym kryterium niezbędnym do rozpoznania zespołu Panayiotopoulosa są napady autonomiczne, najczęściej pod postacią wymiotów oraz stwierdzana w badaniu EEG czynność napadowa o wieloogniskowej i zmiennej lokalizacji (choć najczęściej w okolicy potylicznej) w czasie kolejnych napadów [3, 5, 6, 7].

Zespół Panayiotopoulosa został stosunkowo niedawno wyodrębniony, dlatego początkowo częstość występowania nie była jednoznacznie ustalona [9, 10]. Obecnie jest coraz częściej rozpoznawanym przez neurologów zespołem padaczkowym wieku dziecięcego; częstość szacuje się na 1 na 10 do 1 na 20 dzieci z padaczką, podobną u obu płci [11, 12]. Według Covanis i wsp zespół Panayiotopoulosa dotyczy 13% dzieci w wieku 3-6 lat oraz 6% w wieku 1-15 lat [12].

Początek objawów występuje między 1-14 rokiem życia, ze szczytem miedzy 3-6 rż. (76% przypadków) [3, 12, 13, 14, 15]. Około 5-17% pacjentów z tym zespołem padaczkowym ma dodatni wywiad w kierunku drgawek gorączkowych [3]. Na początku napadu lub w okresie poprzedzającym napad mogą pojawić się zmiany zachowania, które polegają na niepokoju, czasami wręcz pobudzeniu ruchowym bądź też nadmiernym uspokojeniu i wycofaniu się dziecka [16]. Pacjent może skarżyć się na gorsze samopoczucie, nudności, ale w tym czasie jest przytomny i pozostaje w logicznym kontakcie, wobec czego doszukiwanie się w tych objawach napadu padaczkowego jest niezwykle trudne i może być mylące. Następnie zaczynają się wymioty, niejednokrotnie obfite, mogące trwać do kilku godzin, prowadząc czasami do odwodnienia [17, 18].

Do napadów autonomicznych koniecznych do rozpoznania zespołu Panayiotopoulosa należą: nudności,wymioty, hipersaliwacja, sinica, bladość powłok skórnych, zaburzenia termoregulacji, bóle głowy, zaburzenia rytmu serca i oddechu [3,16]. Wymioty występują u około 80% pacjentów i są najczęstszym, często pierwszym objawem napadu. Objawy autonomiczne mogą być poprzedzone bólami głowy lub z nimi współistnieć. Częstym objawem towarzyszącym wymiotom jest jednostronny zwrot gałek ocznych w bok wraz ze zwrotem głowy w tę samą stronę oraz towarzyszące zaburzenia świadomości [16, 17]. W części przypadków z objawami autonomicznymi mogą współwystępować również objawy z wieczka czołowo-ciemieniowego, takie jak: ogniskowe drgawki kloniczne w obrębie twarzy lub języka, zaburzenia artykulacji, automatyzmy pod postacią żucia, przełykania, ślinienia oraz halucynacje smakowe, lęk [3]. U 20% pacjentów (zazwyczaj u pacjentów, u których nie włączono żadnego leczenia p/padaczkowego), dziecięca padaczka z wyładowaniami w okolicy potylicznej o wczesnym początku poprzedza występowanie innych zespołów, najczęściej dziecięcej padaczki z iglicami w okolicy centralno-skroniowej. Z tego względu w różnicowaniu należy uwzględnić również inne zespoły wieku dziecięcego m.in. dziecięcą padaczkę z iglicami w okolicach centralno-skroniowej (CECTS) oraz atypową dziecięcą padaczkę z iglicami w okolicy centralno skroniowej (ACECTS). W CECTS nigdy nie występują uogólnione napady drgawkowe w czuwaniu, zwykle nie występują objawy autonomiczne, co wyklucza to rozpoznanie u naszej pacjentki. W ACECTS charakterystyczne jest występowanie poza nocnymi napadami ogniskowymi i napadami z wieczka czołowo-ciemieniowego również atypowych napadów nieświadomości w czuwaniu [3].

Nudności i wymioty występujące u pacjentów z zespołem Panayiotopoulosa często początkowo nie są traktowane, jako element napadu padaczkowego, ale sugerują podejrzenie zapalenia żołądka i jelit, choroby lokomocyjnej bądź migreny, alergii pokarmowej, natomiast dołączenie się napadów połowiczych i uogólnionych nasuwa podejrzenie zapalenia mózgu lub procesu rozrostowego w ośrodkowym układzie nerwowym [5, 12].

W diagnostyce różnicowej u przedstawionej pacjentki wzięto pod uwagę wszystkie powyższe przyczyny, jak również uwzględniono dziecięce zespoły epizodyczne, takie jak: zespół cyklicznych wymiotów, migrenę brzuszną i przedsionkową, łagodne napadowe zawroty głowy, łagodny napadowy kręcz szyi [3]. Pacjentka nie spełniała kryteriów ich rozpoznania.

Wynik badania neurologicznego pacjentów z zespołem Panayiotopoulosa jest prawidłowy. Choroba najczęściej nie powoduje zaburzeń rozwoju psychoruchowego oraz intelektualnego dziecka. Czasami w okresie nasilenia napadów u części dzieci mogą wystąpić niewielkie zaburzenia funkcji wykonawczych i językowych [3].

W zapisach EEG międzynapadowych występują zmiany napadowe wieloogniskowe pod postacią iglic i zespołów iglicy z falą wolną o zmiennej lokalizacji w kolejnych badaniach. U 75% pacjentów z zespołem Panayiotopoulosa w badaniu rejestrowano zmiany w okolicy potylicznej, u 25% zmiany w okolicy innej niż potyliczna, tylko u 2% występują wyładowania uogólnione, natomiast u 9% nie stwierdzono zmian napadowych [4, 16, 19]. W retrospektywnym badaniu Caraballo z 2015 r., w badaniu

EEG stwierdzono uogólnione wyładowania zespołów iglicy z falą wolną, zarówno w zapisach wykonanych w stanie czuwania, jak i we śnie, które były na początku jedyną manifestacją zespołu [20]. W zdecydowanej większości przypadków nie występują zmiany w badaniach obrazowych głowy [3]. Napady ogniskowe ewoluują do obustronnych napadów toniczno-klonicznych u 21-36% dzieci [12, 21]. U 2/3 pacjentów objawy zaczynają się w czasie snu, często podczas podróży [17]. Zwykle trwają one powyżej 6 minut, choć wykazują tendencję do przedłużania się [3]. Częstość występowania stanu padaczkowego jest zróżnicowana i waha się od 4% do nawet 30% [10, 17]. Napady zwykle ustępują po 11-13 rż. [22], po 2-4 latach od pierwszego napadu, zwykle przed 16 rokiem życia [21]. Nie stwierdzono ryzyka nawrotu napadów w wieku dorosłym [3]. Zazwyczaj czas od wystąpienia pierwszego napadu do włączenia leczenia waha się od 1 miesiąca do 3 lat [16].

Zespół Panayiotopoulosa uważa się za padaczkę zwykle niewymagającą leczenia p/padaczkowego, napady mają tendencję do ustępowania [3], ale opisano również ciężkie przypadki wystąpienia niewydolności krążeniowo-oddechowej. Mujawar i wsp. opisali przypadek 3,5-letniego dziecka z zespołem Panayiotopoulosa, u którego w czasie napadu doszło do niewydolności krążeniowooddechowej wymagającej resuscytacji [23]. Podobny przypadek opisał Dirani i wsp. – dziewczynki 4-letniej, dotychczas zdrowej, u której pierwszym objawem były: wymioty, prawostronny zwrot gałek ocznych oraz bladość, a w kolejnych minutach niewydolność oddechowa wymagająca intubacji [24]. Zatem warto podkreślić, że choć rzadko, ale możliwy jest ciężki przebieg choroby, łącznie ze stanami zagrażającymi życiu.

Za rozpoznaniem zespołu Panayiotopoulosa u omawianej pacjentki przemawia: typowy wiek występowania objawów, typowe objawy autonomiczne pod postacią: wymiotów, bólu głowy, zwrotu gałek ocznych, zaburzeń świadomości (w naszym przypadku dziewczynka była „jakby nieobecna”), lęku oraz głośnego przełykania śliny, zmiany w EEG (w odprowadzeniach potylicznych czynność napadowa o charakterze iglic), prawidłowy rozwój dziecka oraz efekt kliniczny zastosowanego leczenia p/padaczkowego. Zespół Panayiotopoulosa jest chorobą o łagodnym przebiegu, zwykle niezaburzającą prawidłowego rozwoju psychoruchowego, dobrze znaną neurologom, ale jeszcze słabo pediatrom.

## Wnioski

Nawracające wymioty u dzieci mogą być objawem różnych schorzeń, w tym padaczki.

Wystąpienie zaburzeń orofaryngealnych i uogólnionych drgawek w trakcie hospitalizacji w Klinice pozwoliło na ustalenie właściwego rozpoznania.

## Skróty/Abbreviations

EOCOE: Early onset childhood occipital epilepsy
CECTS: Childhood epilepsy with centrotemporal spikes
ACECTS: Atypical childhood epilepsy with centrotemporal spikes
